# Butin Mitigates Memory Impairment in Streptozotocin-Induced Diabetic Rats by Inhibiting Oxidative Stress and Inflammatory Responses

**DOI:** 10.3390/metabo12111050

**Published:** 2022-11-01

**Authors:** Asma B. Omer, Mahmood Hassan Dalhat, Mohammad Kaleem Khan, Obaid Afzal, Abdulmalik S. A. Altamimi, Sami I. Alzarea, Waleed Hassan Almalki, Imran Kazmi

**Affiliations:** 1 Department of Basic Health Sciences, Foundation Year for the Health Colleges, Princess Nourah bint Abdul Rahman University, Riyadh 11671, Saudi Arabia; 2 Department of Biochemistry, Faculty of Science, King Abdulaziz University, Jeddah 21589, Saudi Arabia; 3 Department of Pharmacology, Dadasaheb Balpande College of Pharmacy, Nagpur 440037, India; 4 Department of Pharmaceutical Chemistry, College of Pharmacy, Prince Sattam bin Abdulaziz University, Al-Kharj 11942, Saudi Arabia; 5 Department of Pharmacology, College of Pharmacy, Jouf University, Sakaka 72341, Saudi Arabia; 6 Department of Pharmacology, College of Pharmacy, Umm Al-Qura University, Makkah 21955, Saudi Arabia

**Keywords:** butin, flavonoids, acetylcholinesterase, neuroprotective, streptozotocin

## Abstract

It has been reported from the previous literature that butin restores mitochondrial dysfunction by modulation of oxidative stress and glutamate-induced neurotoxicity in mouse hippocampus HT22 cells. Butin also possesses an anti-Huntington’s effect in rats. Considering the current background, this study was designed to evaluate the neuroprotective effect of butin against memory loss caused by streptozotocin (STZ). STZ (40 mg/kg) was intraperitoneally injected into rats. Three days later, diabetic rats were identified and included in the study. A total of 30 rats (12 nondiabetic and 18 diabetics) were grouped as Group A (control-non-diabetic rats) and Group B (STZ diabetic control) were treated with 1 mL of sodium CMC (0.5% *w/v*). Group C (STZ+ butin 25) were treated with butin 25 mg/kg. Group D (STZ+ butin 50) and Group E (butin per se) were administered with butin 50 mg/kg. Each therapy was administered orally once each day for 15-day. The Morris water maze and the Y-maze behavioural tests were run throughout the experimental programme. Animals were put to death on day 15 and their brains were removed for biochemical assays (CAT, SOD, GSH, MDA, nitrite, acetylcholinesterase (AchE), IL-1, and mitochondrial enzyme complexes). Rats with neurobehavioral impairments brought on by STZ have less spontaneous movement, learning capacity, and memory. Additionally, STZ decreased endogenous antioxidants and increased pro-inflammatory cytokines, nitrite, MDA, and AchE. Neurobehavioral deficits and metabolic markers were dramatically improved by butin.

## 1. Introduction

In the twenty-first century, neurodegenerative disorders (NDs) have grown to be a significant public health concern [1,2]. NDs are becoming more common over the world as the elderly population grows [1,2]. Presently there is no effective treatment available for ND; hence, the research on the treatment of neurodegenerative illnesses is becoming increasingly important as the world’s population ages [1,2,3]. The World Health Organization predicts that by 2050, there will be approximately 2 billion individuals over the age of 60 in the world, up from a current ratio of 12 per cent in 2015 [2]. 

Alzheimer’s disease (AD) causes long-term learning and cognitive problems [4]. It is believed that the development of AD is influenced by the advancement of neurodegenerative processes in the hippocampus, such as microglia activation, inflammation of neurons, oxidative stress, loss of metabolic energy, and neuronal degeneration [4]. There is overwhelming proof that diabetes mellitus (DM) harms the central nervous system [5]. In diabetics and in animals, neurological diseases such as anxiety, sadness, dementia and AD are seen [6]. The key hallmarks associated with DM include oxidative stress and inflammation, as well as altered cell signalling pathways, and these are the leading known mechanisms responsible for the brain-damaging consequences of DM [6].

The diabetogenic drug streptozotocin (STZ) causes Amyloid-β (Aβ) to build up in the brains of treated diabetic animals. Additionally, hyperglycaemia exacerbated memory loss owing to cerebrovascular inflammation and deposition in the AD animal model. Similar processes underlie the majority of neurodegenerative diseases, including elevated oxidative stress, ongoing neuroinflammation, protease-resistant misfolding, and the formation of aggregated proteins [2,7,8].

New disease-modifying medications that can deliberate down or arrest the development of neurodegenerative diseases have been created recently due to the increased interest in medicinal plants and chemicals derived from plants [2,9,10]. Many medicinal plants and plant-based products were tested for different neurodegeneration models in animals [9,11,12]. 

According to a review of the literature, some herbal products have been claimed to have therapeutic actions in preclinical neurodegeneration models [11,12,13,14,15]. 

Foods rich in flavonoids are beneficial for animals with dementia and cognitive impairment [16]. In brain cells, flavonoids successfully reduce oxidative stress and inflammation [17].

**Figure 1:**
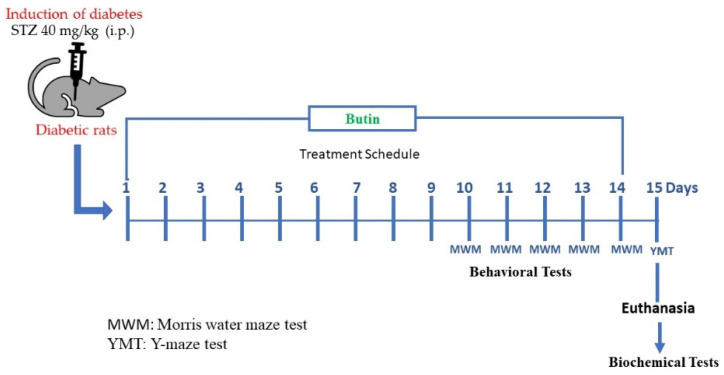
Scheme of experimental design.

Butin (7,3′,4′-trihydroxydihydroflavone) is a flavonoid constituent of *Vernonia anthelmintica*, *Acacia mearnsii*, and *Dalbergia odorifera* (Figure 1) [18,19]. Strong antioxidant [20], anti-inflammatory [21], and antiplatelet activity [21] are all properties of butin. Butin protects mouse HT22 hippocampus cells from glutamate-induced neurotoxicity [19,22]. Furthermore, butin reduces brain oedema in experimental rats [21] and guards against dysfunction of mitochondrial brought by oxidative stress [19]. Butin has been demonstrated to have a protective effect in diabetic mice against ischemia/reperfusion-induced heart damage [23]. More recently, butin has been reported for its neuroprotective actions against chemically induced Huntington’s symptoms in rats [24]. These factors made it tempting to investigate butin’s potential protection against rats’ memory deficits brought on by streptozotocin. 

## 2. Material and Methods

### 2.1. Chemicals and Reagents

STZ, analytical kits for TNF-α, IL-6 and IL-1β were analysed by rat enzyme-linked immunosorbent assay kit (Sigma-Aldrich, St. Louis, MO, USA).

### 2.2. Animals

Male Wistar rats (180 ± 20 g) were maintained at 21 °C, 61 ± 2% humidity and 12 h light/dark cycle. All rats had unfettered access to standard feed and water during the trial. The ethics committee approved all of the study’s protocols (TRS/PT/021/009).

### 2.3. Experimental Design

To cause diabetes, 40 mg/kg (i.p.) of STZ diluted in citrate buffer (0.5 M, pH 4.5) was injected into animals [6]. Butin was diluted with sodium CMC (0.5% *w/v*) and given orally for 15 days [24].

#### 2.3.1. Induction of Diabetes Mellitus 

DM was induced in rats as per previously published protocol [6]. Briefly, a dose of STZ solution (40 mg/kg) was injected (i.p.) into the rats and 5% of glucose solution was given orally. Levels of blood glucose were assessed three days after STZ administration. Animals with blood glucose >300 mg/dL were classified as diabetes and included in the trial to determine the effectiveness of butin against STZ-induced memory deficits in diabetic rats.

#### 2.3.2. Evaluation of Butin against STZ-Induced Memory Deficit in Diabetic Rats

A total of 30 rats (12 nondiabetic and 18 diabetics) were grouped: Group A (control, nondiabetic rats) and Group B (STZ control rats) were treated with 1 mL of sodium CMC (0.5% *w/v*), these groups were served as normal control and STZ control, respectively. Group C (STZ + butin 25) were treated with 25 mg/kg of butin. Group D (STZ + butin 50) and Group E (butin per se) were treated with 50 mg/kg of butin. Groups C and D served as test groups and Group E served as butin control group. All the above-mentioned treatments were given once a day orally for 15 days. At the end of the experiment, blood glucose levels were monitored. Animal behavioural tests were undertaken throughout the experimental period. Animals were slaughtered on day 15 and their brains were extracted for biochemical assays after behavioural measures. The scheme of experimental design is shown in Figure 1. 

### 2.4. Behavioural Tests

#### 2.4.1. Morris Water Maze (MWM) Test

Animals were taught every day for three trials each day, spaced 120 s apart, 120 s was kept as the cut-off time throughout the MWM evaluation period to prevent stress on the animals. During the training session, if the rat could not locate a concealed platform in 120 s, it was assisted onto a platform. On days 10 to 14 following the training sessions, the animals’ spatial memory and learning capacity were assessed. Day 15 saw the removal of the hidden platform and the execution of a probing test 24 h after phase of acquisition. The TSTQ, which previously had a hidden platform, was observed after animals were let to swim for 120 s. The length of TSTQ revealed how much memory consolidation had happened following learning [25,26].

#### 2.4.2. Y-Maze Test

Three identical arms made up the Y-maze (45 × 12 × 35 cm) that diverged at a 120° angle from one another and a central equilateral triangular region. Eight minutes were given for each animal to inspect itself after it was put in the centre of the arena. Rats prefer to investigate the arm that has not been explored in a while; thus, they alternate excursions between the three arms. Animals need to employ working memory for efficient alternation; therefore, they should keep track of arms that were most recently visited, and such information is updated regularly. The placement of the rat’s four paws inside that arm was rated as an arm entry. As dependent variables, it was also noticed that number of arm entries, number of triads and the percentage of an alternation. Three consecutive selections into three different arms were used to define an alternation. The total number of arm entries minus two was used to compute the maximum number of alternations that could be made. The low proportion of alternation shows weak spatial working memory and, consequently, less spontaneous alternation because the rat cannot recall which arm it visited last [27].

### 2.5. Biochemical Tests

#### 2.5.1. Brain Tissue Homogenisation

Isotonic saline was used to cool and clean the brains. The phosphate buffer was used to homogenise the whole brain samples (0.1 M, pH 7.4, ice-cold). After centrifuging the homogenate, the biochemical makeup of the supernatant was evaluated [9,12,28].

#### 2.5.2. Acetylcholinesterase (AchE) Activity

To assess AchE activity a technique similar to that published by Ellman et al. (1961) was used. The 3 mL sodium phosphate buffer (0.01 M, pH 8), 0.10 mL of acetylthiocholine iodide and 0.10 mL of DTNB (Ellman’s reagent) and 0.05 mL of supernatant are the contents of assay mixture. The absorbance changes were measured at 412 nm immediately. The enzymatic activity was expressed as μM/mg protein [29]. 

#### 2.5.3. Endogenous Antioxidants

The amount of GSH was measured using the Ellman method [30]. SOD was measured using the Misra and Frodvich technique, the supernatant of brain homogenate (0.2 mL) was mixed with 0.8 mL 50 mM glycine buffer (pH 10.4). By adding 0.02 mL of epinephrine in it initiated the reaction. After 5 min, the absorbance was measured at 480 nm [31]. To measure catalase activity, 0.1 mL of supernatant was added to 1.9 mL phosphate buffer (pH 7.0, 50 mM) in the cuvette. An amount of 1.0 mL of freshly prepared H_2_O_2_ (30 mM) was added to initiate the reaction. The catalase activity was represented as μM/H_2_O_2_ decomposed/min [32].

#### 2.5.4. Markers of Oxidative and Nitrative Stress 

The Wills method was used to calculate malondialdehyde (MDA) in brain homogenate. The MDA content was measured at 532 nm after its reaction with thiobarbituric acid. The MDA concentration was measured in nanomoles per mg of protein [33]. Nitric levels were measured by measuring the amount of nitrite using the Griess reagent. The 0.1 mL of supernatant was added to 0.5 mL of Griess reagent (0.1% N-(1-naphthyl) ethylenediamine dihydrochloride, 1% sulphanilamide and 2.5% phosphoric acid), and the absorbance was measured at 546 nm. The amount of nitrite was calculated using a sodium nitrite standard curve, and the results were represented in nM/mg protein [15,34].

#### 2.5.5. Pro-Inflammatory Cytokines

Pro-inflammatory cytokines such as IL-1 were measured using immunoassay kits. Marker concentrations were calculated using standard curves and expressed as pg/mL protein.

#### 2.5.6. Estimation of Mitochondrial Complex I, II and III

Mitochondrial complex I, II and III were quantitatively measured as per the experimental protocol published elsewhere [35].

### 2.6. Statistical Analysis

Graph Pad Prism was used to do the statistical analysis. The data are presented as S.E.M. For Morris water maze test: two-way ANOVA followed by Bonferroni test; for all other parameters: one-way ANOVA followed by Tukey’s test. *p* < 0.05 was used as the significance level.

## 3. Results

### 3.1. Blood Glucose Levels

**Figure 2:**
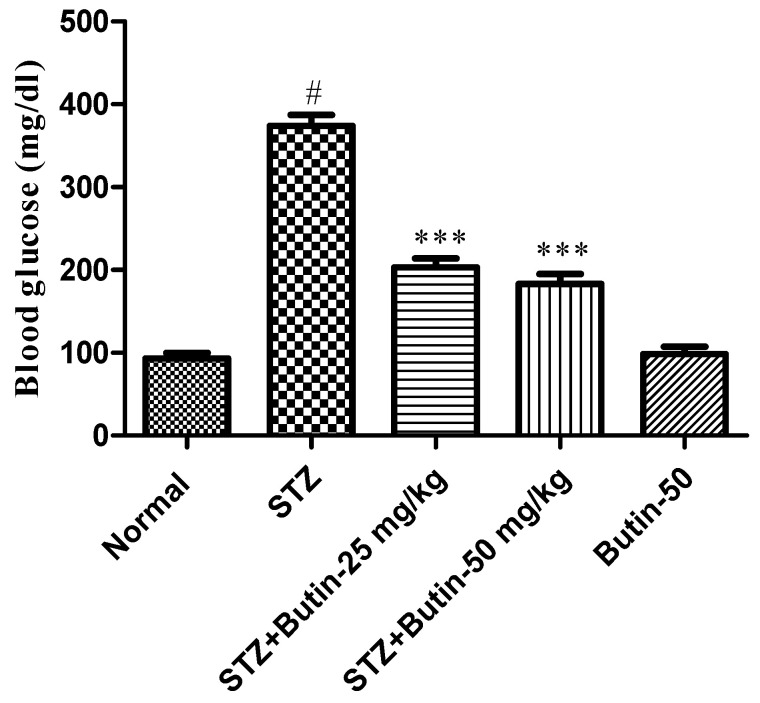
Effect of butin on blood glucose in STZ-treated diabetic rats. Mean ± S.E.M. (n = 6), ^#^ *p* < 0.05 vs. normal control, *** *p* < 0.001 vs. STZ control. One-way ANOVA followed by Tukey’s test.

When compared to STZ-induced animals, the control had a substantial (*p* < 0.001) rise in blood glucose levels. Furthermore, the butin (10, and 20 mg/kg) group treatment of STZ-treated rats was significant reductions in blood glucose as compared to the STZ control group (*p* < 0.001). The blood glucose level in butin per se rats treated did not significantly change (Figure 2).

### 3.2. Behavioural Parameters

#### 3.2.1. MWM

**Figure 3:**
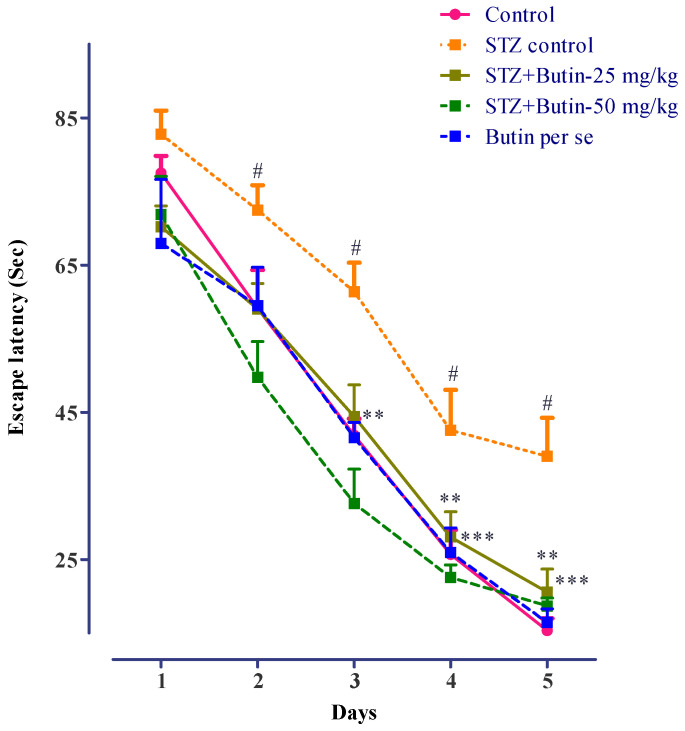
Effect of butin on Morris water maze test in STZ-treated diabetic rats. Mean ± S.E.M. (n = 6), ^#^ *p* < 0.05 vs. normal control, ** *p* < 0.01 vs. STZ control and *** *p* < 0.001 vs. STZ control. Two-way ANOVA followed by Bonferroni test.

STZ impaired the memory of rats and increased latency to escape from swimming and enter the immovable platform in the MWM test. The escape latency of STZ-treated rats was increased on day 3 (*p* < 0.01), day 4 (*p* < 0.01) and day 5 (*p* < 0.001) as compared normal. Butin treatment of STZ-treated rats reduced their memory deficit, as evidenced by decreased latency in finding an immovable platform in the MWM. On days 3, 4 and 5, were significant (*p* < 0.01) vs. STZ control rats. Butin per se to nondiabetic animals did not produce significant changes vs. normal control animals. Figure 3 depicts the detailed findings of the MWM test.

#### 3.2.2. Y-Maze Test

**Figure 4:**
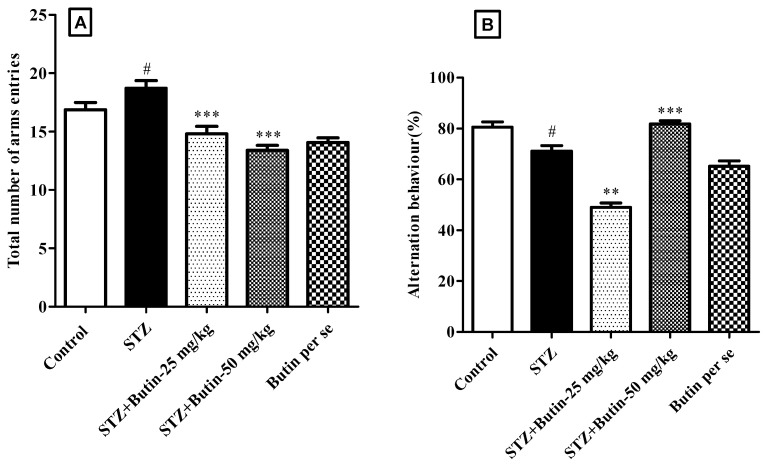
Effect of butin on Y-maze test in STZ-treated diabetic rats. (**A**) Total number of arm entries and (**B**) spontaneous alternation. Mean ± S.E.M. (n = 6), ^#^ *p* < 0.05 vs. normal control, ** *p* < 0.01 vs. STZ control and *** *p* < 0.001 vs. STZ control. One-way ANOVA followed by Tukey’s test.

When STZ animals were compared to control rats, total arm entries and spontaneous alternation were lower (*p* < 0.05). Butin to STZ-treated rats restored total arm entries (*p* < 0.001) and spontaneous alternation (*p* < 0.001) vs. STZ control animals. Administrations of butin per se (50 mg/kg) to nondiabetic animals do not produce significant changes vs. normal control animals (Figure 4). 

### 3.3. Biochemical Parameters

#### 3.3.1. AchE Activity

**Figure 5:**
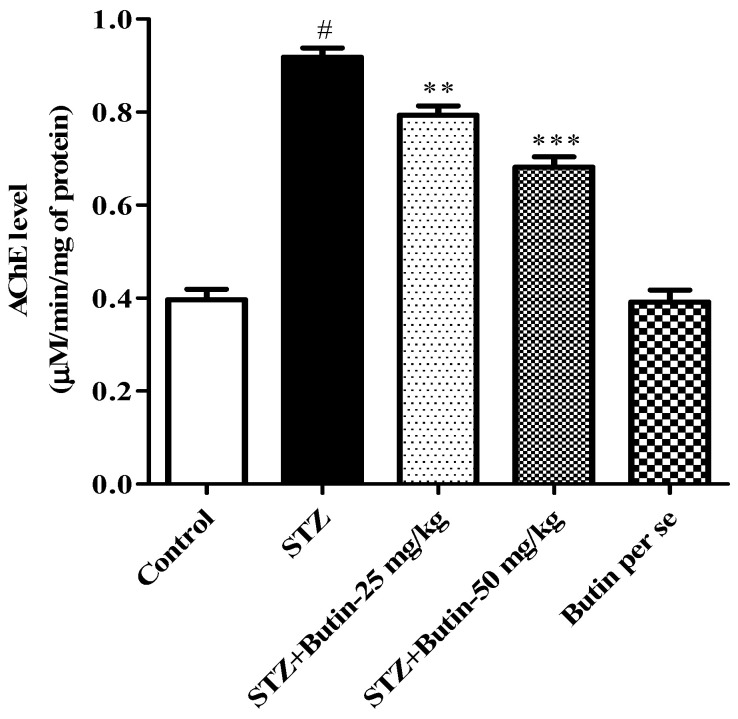
Effect of butin on Acetylcholinesterase activity in STZ-treated diabetic rats. Mean ± S.E.M. (n = 6), ^#^ *p* < 0.05 vs. normal control, ** *p* < 0.01 vs. STZ control and *** *p* < 0.001 vs. STZ control. One-way ANOVA followed by Tukey’s test.

The STZ group had more than two-fold greater AchE levels (*p* < 0.05) than the control group. Butin 25 and 50 mg/kg (*p* < 0.01; *p* < 0.001) significantly decreased AchE levels by 13.61–25.77% in STZ treated animals. AchE activity in butin per se rats treated did not significantly change. Figure 5 depicts the outcome of the AchE estimation.

#### 3.3.2. Endogenous Antioxidants

**Figure 6:**
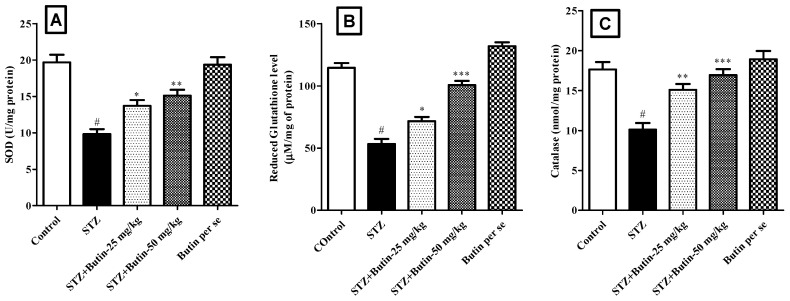
Effect of butin on endogenous antioxidant status in STZ-treated diabetic rats. (**A**) Superoxide dismutase, (**B**) reduced glutathione and (**C**) catalase. Mean ± S.E.M. (n = 6), ^#^ *p* < 0.05 vs. normal control, * *p* < 0.05 vs. STZ control ,** *p* < 0.01 vs. STZ control and *** *p* < 0.001 vs. STZ control. One-way ANOVA followed by Tukey’s test.

In animals treated with STZ, endogenous antioxidants (SOD, GSH, and catalase) levels were disturbed. STZ control animals had considerably (*p* < 0.05) lower levels of SOD (50.11%), GSH (53.39%), and catalase (42.68%) than normal control rats. Treatment with butin 25 and 50 mg/kg to STZ injected animals, restored GSH (*p* < 0.05 and *p* < 0.001), SOD (*p* < 0.05 and *p* < 0.01) and catalase (*p* < 0.01 and *p* < 0.001) levels. Administration of butin per se to non-diabetic rats slightly elevated all the estimated endogenous antioxidants (Figure 6). 

#### 3.3.3. Markers of Oxidative and Nitrative Stress 

**Figure 7:**
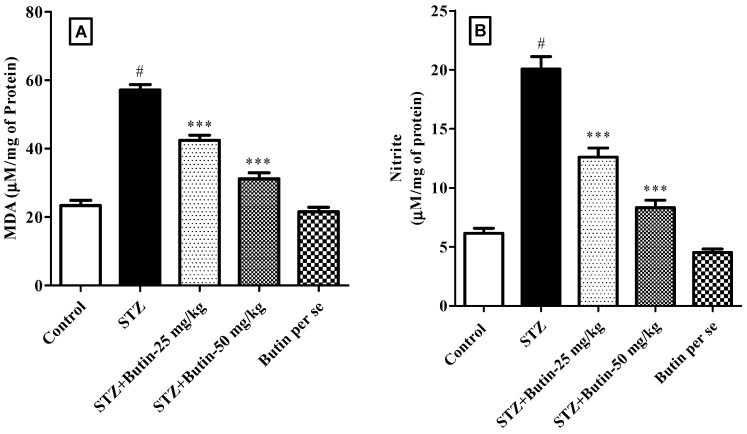
Effect of butin on (**A**) malondialdehyde and (**B**) nitrite levels in STZ-treated diabetic rats. Mean ± S.E.M. (n = 6), ^#^ *p* < 0.05 vs. normal control and *** *p* < 0.001 vs. STZ control. One-way ANOVA followed by Tukey’s test.

MDA (144.33%) and nitrite (226.34%) levels in STZ control rats were greater than those in the control (*p* < 0.05). Butin treatment to STZ-injected animals attenuated the increased levels of MDA (25.75–45.42%) and nitrite (37.20–58.47%). The results were statistically significant with a *p*-value of 0.001 when compared to STZ control animals. Butin per se (50 mg/kg) in nondiabetic animals did not produce significant changes. Figure 7 depicts the MDA and nitrite levels as a result. 

#### 3.3.4. Pro-Inflammatory Cytokines

**Figure 8:**
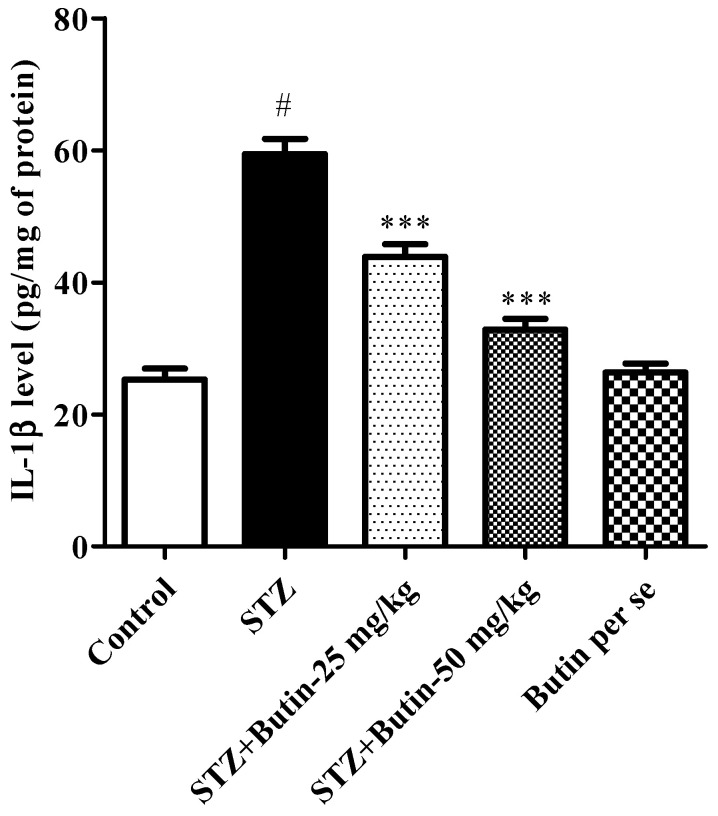
Effect of butin on pro-inflammatory cytokine interleukin-1β in STZ-treated diabetic rats. Mean ± S.E.M. (n = 6), ^#^ *p* < 0.05 vs. normal control and *** *p* < 0.001 vs. STZ control. One-way ANOVA followed by Tukey’s test.

Rats treated with STZ had considerably (*p* < 0.05) higher pro-inflammatory cytokine IL-1β levels than healthy control animals. When butin 25 and 50 mg/kg was administered to STZ-treated rats, the levels of IL-1β were reduced approaching normal (*p* < 0.01 and *p* < 0.01) in contrast to the STZ control group. When compared to the normal control rats, butin per se (50 mg/kg) administration to non-diabetic animals did not result in any appreciable alterations. Figure 8 displays the findings of the IL-1 levels.

#### 3.3.5. Estimation of Mitochondrial Complex I, II and III

**Figure 9:**
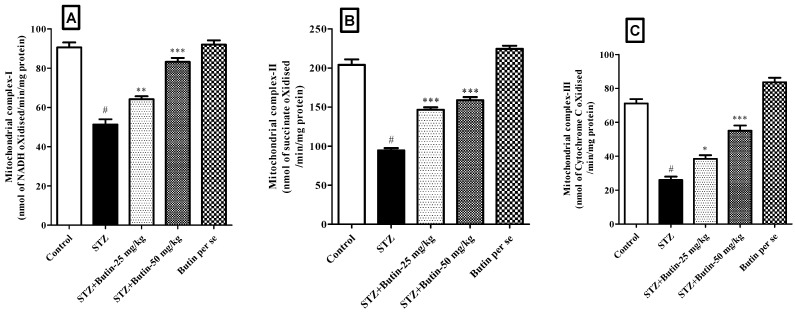
Effect of butin on (**A**) mitochondrial complex I, (**B**) mitochondrial complex II and (**C**) mitochondrial complex III levels in STZ-treated diabetic rats. Mean ± S.E.M. (n = 6), ^#^ *p* < 0.05 vs. normal control, * *p* < 0.01 vs. STZ control, ** *p* < 0.01 vs. STZ control and *** *p* < 0.001 vs. STZ control. One-way ANOVA followed by Tukey’s test.

When compared to the STZ control group led to a significantly (*p* < 0.05) decreased level of the mitochondrial complex I, II, and III enzymes. The levels of mitochondrial complex I (*p* < 0.01 and *p* < 0.001), II (*p* < 0.001 and *p* < 0.001), and III (*p* < 0.05 and *p* < 0.001) enzymes were reduced by butin 25 and 50 mg/kg toward the baseline. When compared to the normal control animals, butin per se (50 mg/kg) to non-diabetic rats did not result in any appreciable modifications in the mitochondrial complex enzymes. Figure 9 displays the outcomes of the mitochondrial complex I, II, and III enzyme levels.

## 4. Discussion

The results of butin on STZ-induced memory impairment were assessed utilising exteroceptive behavioural models such as MWM and Y-maze tests. These activities have been widely utilised to evaluate learning and memory in a variety of animal paradigms, most notably rodent behavioural manipulations [36]. In addition, the effect of butin on STZ-induced oxidative and nitrative stress and proinflammatory cytokines was also assessed. 

The central cholinergic system aids both learning and memory [37] and Ach is a crucial neurotransmitter that influences cognitive function and learning [37,38]. In the synaptic cleft, ACh is released from vesicles and moved. There, it is processed by AChE into acetic acid and choline [39]. On the other side, excessive AChE activity may result in a shortage of ACh and cognitive impairment [40].

In the current investigation, STZ control rats increased blood glucose levels as compared to the normal control group and exhibits significant alterations by using butin. Previously reported that an increase in blood glucose level is an important hallmark for the pathogenesis of AD [41].

STZ injection enhanced AChE levels as associated to control rats. Whereas butin treatment reduced AChE activity compared to STZ-treated animals. These findings are consistent with previously published data [24] and point to the ability of butin to protect treated rats from STZ-induced AChE activity.

The spontaneous alternation score and total arm entries in the Y-maze test are used to assess working memory [36]. In this investigation, the STZ control rats had fewer total arm entries and spontaneous arm alternations than the normal control animals. This demonstrates that animals given STZ have weak working memory. Butin treatment increased total arm entries by 44.56–71.56% and spontaneous alternation by 21.53–39.41% in STZ-treated rats. These data show that butin protects treated rats from STZ-induced memory loss and the results are parallel with the neuroprotective effect of butin in 3-NP-treated rats [24].

The MWM test can be used to assess long-term spatial memory and learning ability [37,42,43]. STZ therapy resulted in longer escape latency, indicating impaired spatial memory and learning abilities in the animals. Butin treatment of STZ-treated rats improved escape latency in the MWM test. These findings shows the protective effect of butin against the STZ-induced memory deficits in rats. 

STZ injections may result in oxidative stress, inflammation, and decreased hippocampus synaptic transmission [44]. The results of the current investigation supported the ones mentioned before. It was shown that STZ administration to rats significantly increased oxidative and nitrative stress by increased ROS production, increased nitrite and MDA levels, and decreased levels of endogenous antioxidants in treated rats. When levels of peroxides and reactive oxygen species (ROS) exceed natural antioxidant defences, initiates oxidative stress-mediated neuronal insults. In the brain, lipid peroxidation (LPO) attacks polyunsaturated fatty acids [14]. The brain is also more susceptible to oxidative injury because it has few antioxidant defence mechanisms [13,14]. 

Butin usage reduced levels of nitrate and MDA while enhancing endogenous antioxidant status. These outcomes are in line with the results of the studied protective effect of butin against ischemia/reperfusion-induced myocardial injury in diabetic mice. In this study, it is reported that butin enhances activities in antioxidant enzymes (GSH-Px, GSH, SOD, CAT and GR) and decreases the levels of MDA and ROS in heart tissues, and further study also showed that GSH-Px, GSH, SOD, CAT and HO-1 at both mRNA and protein levels were increased in H9c2 cells. Based on these, it can be postulated that butin may have protective effects in rats with STZ-induced neurotoxicity because of its antioxidant characteristics.

Cellular metabolic alterations in the brains of persons with AD can be noticed early on in the disease, typically before amyloid plaques and neurofibrillary tangles appear [45]. In AD, numerous metabolic pathways are dysfunctional [46], influencing nerve and peripheral cell types [47]. The development and progression of AD appear to be significantly influenced by defects in mitochondrial function, particularly in how they regulate oxidative phosphorylation [45]. The production of ATP is carried out by the electron transport chain [48]. The F0F1-ATP synthase (complex V) enzyme utilises the membrane potential produced by complexes I–IV to produce ATP from ADP and phosphate. complexes I–IV are connected to the respiratory chain’s complexes I–IV [45]. In this study administration of STZ to rats resulted in decreased levels of complex I–III in the brain. When compared to STZ control animals, the administration of butin normalised the levels of mitochondrial complex enzymes. This finding indicates the mitochondrial protection potential of butin against STZ-induced toxicity in rats.

Microglia and astrocytes secrete cytokines and chemokines, which play a significant part in neuroinflammation in AD and cause them to become overactive [49]. Anti-inflammatory therapies can lessen AD pathology in animal transgenic AD models [49]. These findings offer compelling proof that neuroinflammation contributes significantly to the onset of AD. The answer to treating AD may be to reduce neuroinflammation [50,51]. Additionally, the connection between inflammation and the cognitive impairment brought on by STZ has long been known [52]. Cognitive difficulties brought on by STZ treatment will be remedied if excessive inflammation stimulation is treated [49]. 

The results of the current investigation corroborated the aforementioned data by demonstrating that butin decreased the rise in IL-1β levels caused by STZ exposure. These findings suggest that butin’s capacity to decrease neuroinflammation by modifying glial activity underlies its protective benefits against STZ-induced AD-like features [49].

## 5. Conclusion

In conclusion, by reducing oxidative stress and excessive inflammation, butin could counteract STZ-induced memory deficits in diabetic rats. Additionally, butin shields the mitochondrial enzyme complexes from the toxicity brought on by STZ. However, additional investigation is required to discover how butin affects people with memory issues.

## Data Availability

Not applicable.
